# Tumor Regression in HCC Patient with Portal Vein Tumor Thrombosis after Intraportal Radiofrequency Thermal Ablation

**DOI:** 10.1155/2016/6843121

**Published:** 2016-08-08

**Authors:** Malkhaz Mizandari, Tamta Azrumelashvili, Natela Paksashvili, Nino Kikodze, Ia Pantsulaia, Nona Janikashvili, Tinatin Chikovani

**Affiliations:** ^1^Department of Interventional Radiology, Tbilisi State Medical University, High Technology University Clinic, 0144 Tbilisi, Georgia; ^2^Department of Immunology, Tbilisi State Medical University, 0177 Tbilisi, Georgia

## Abstract

Hepatocellular carcinoma (HCC) is the third leading cause of cancer-related death worldwide. Portal vein tumor thrombosis (PVTT) is a frequent entity in HCC, which strictly limits the gold standard treatment options such as surgical resection and transarterial chemoembolization. Therefore, the prognosis of patients with PVTT is extremely poor and an emergence of seeking an alternative option for intervention is inevitable. We present a case of a 60-year-old male patient with HCC induced PVTT who was subjected to the intraportal RFA and stenting-VesOpen procedure. No additional medical intervention was performed. The repeated CT performed 5 months after the VesOpen procedure revealed significant decrease of the tumor size, patent right, and main portal vein and a recanalization of the left portal vein, which was not processed. At this time point, liver functional tests, appetite, and general condition of the patient were improved evidently. This report designates the RFA as an instrumental option of therapeutic intervention for HCC patients with PVTT.

## 1. Introduction

Hepatocellular carcinoma (HCC) is the most common primary liver cancer, the sixth most common cancer overall, and the third most common cause of cancer-related death worldwide [1–3]. Classical treatments for HCC include surgical resection, liver transplantation, and local ablative therapy [4, 5]. Liver transplantation is theoretically the best therapeutic choice, however, limited by the shortage of donor organs, and hepatectomy is considered the standard treatment for patients with preserved liver function [6, 7].

Portal vein thrombosis (PVT) is a common complication of HCC. The management of HCC patients with PVT is more challenging than the ones without PVT [8]. The presence of PVTT limits standard treatment options: liver transplantation and curative resection [9, 10]. Transarterial chemoembolization (TACE) is associated with an increased risk of ischemic liver necrosis in such cases and is, therefore, subjective to a select group of patients with good hepatic function and adequate collateral circulation around the occluded portal vein [11]. Thus, the prognosis of inoperable cases of HCC with PVTT is extremely poor; the average life span after diagnosis is reported to be 3 to 6 months [12, 13].

Radiofrequency thermal ablation (RFA) may be considered as an attainable method in such condition. Details of the percutaneous PVTT ablation procedure, including its safety and feasibility, are previously described by our team [14, 15].

## 2. Case Presentation

**Figure 1:**
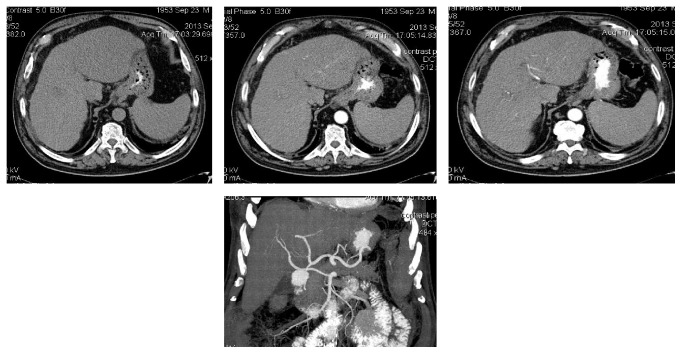
Preprocedure CT (native and arterial phase). Note: superior mesenteric artery type right hepatic artery; left lobe mass main feeder is left hepatic artery.

**Figure 2:**
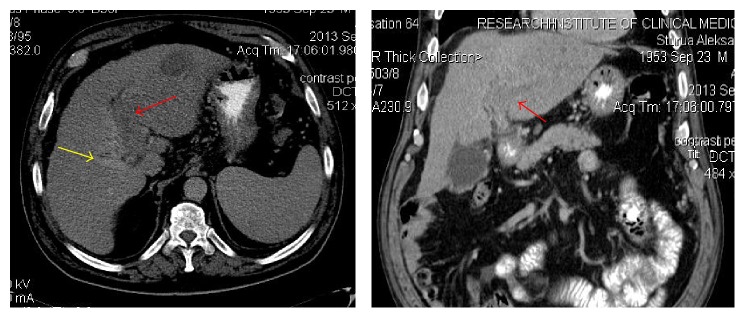
Preprocedure CT (portal phase): RPV patent branch (puncture “target” on VesOpen procedure), yellow arrow; completely obliterated LPV, red arrow.

A 60-year-old man was admitted with three weeks of fatigue and abdominal discomfort. He was documented to suffer from a hepatitis C induced liver cirrhosis (Child-Pugh B) and was admitted to our hospital for US-guided biopsy of the liver mass. CT scan reported a left lobe vascular mass (8-9 cm), with prominent venous phase washout. Sharply circumscribed, hypodense component of 3 cm in size was shown within this mass. Left PV total tumor thrombosis and PV cavernous transformation were also revealed. Thrombus was already protruding into the main PV, so that only right PV remained patent (Figures 1 and 2).

Blood tests revealed severe hypoalbuminemia and thrombocytopenia, moderate hypocoagulation, moderate changes of liver enzymes, and elevated *α*-fetoprotein (Table 1).

The patient first underwent a percutaneous ultrasound (US) guided biopsy of the left lobe mass (from small hypodense component). Morphological study of biopsy specimens revealed the diagnosis of HCC. Based on the existence of a PVTT, this patient was not subjected to the surgical resection and/or TACE procedure. The VesOpen procedure, aiming at normal blood flow restoration to the right PV, has been proven on MDT discussion.

The patient returned to the hospital a month later to undergo portal vein recanalization, via intraportal RFA and stenting-VesOpen procedure.

In VesOpen procedure, the right PV was accessed by 18 G puncture needle using real-time US guidance; contrast injection showed the portography “above” the thrombus, manifesting the PV thrombus “upper” border. 0.035-inch diameter wire was conducted through the thrombus into SMV using 5 Fr guiding catheter and portography “below” the thrombus was performed, documenting the PV thrombus “lower” border. 8 Fr diameter introducer sheath was positioned and 8 Fr endoluminal device (RITA® Model 1500X RF Generator AngioDynamics, EMcision 8F VesOpen 2800) was introduced into the thrombus for 2-session processing. The 14 mm diameter self-expanding vascular stent (Zilver 635® Vascular Self-Expanding Stent | Cook Medical) was positioned into the thrombus and postdilated by balloon. Postprocedure portography showed the main PV patency complete restoration maintaining the normal blood flow into the right portal vein. The VesOpen procedure was completed with working track ablation by the same RF device.

The patient tolerated the procedure well; no intraprocedural complications were detected. On postprocedure follow-up (in 3 hours) fluid in small pelvis (blood) has been detected in 3 hours and as the amount was increasing slightly, small pelvis drainage has been performed and up to 800 cc blood was evacuated. The patient stayed in clinic for 36 hours and received the fresh frozen plasma and red blood cell mass infusion.

After being discharged from hospital, the patient was referred to the hematologist and hepatologist for further consultations and to prepare for a probable TACE.

Patient refused to undergo the TACE and visited the clinic only for the consultations 5 months later. His condition had been improved dramatically; albumin rose to 32 g/dL. Coagulation status and liver functional tests, appetite, and functional status had improved as well.

**Figure 3:**
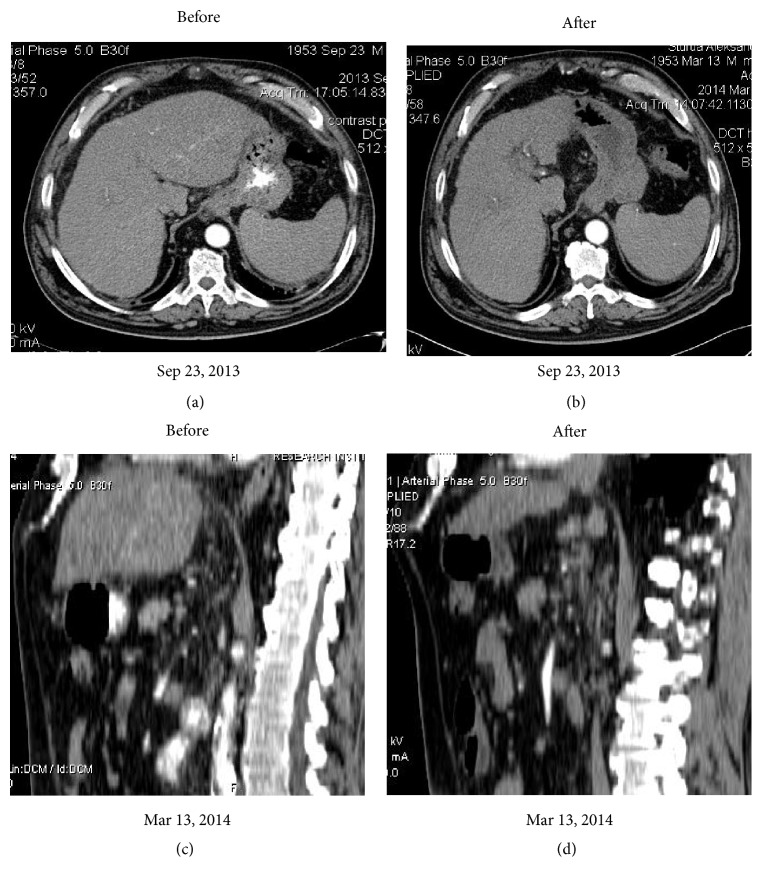
Comparison: CT before and in 5 months after VesOpen procedure. Note the decreased size and normal shape of the left lobe.

**Figure 4:**
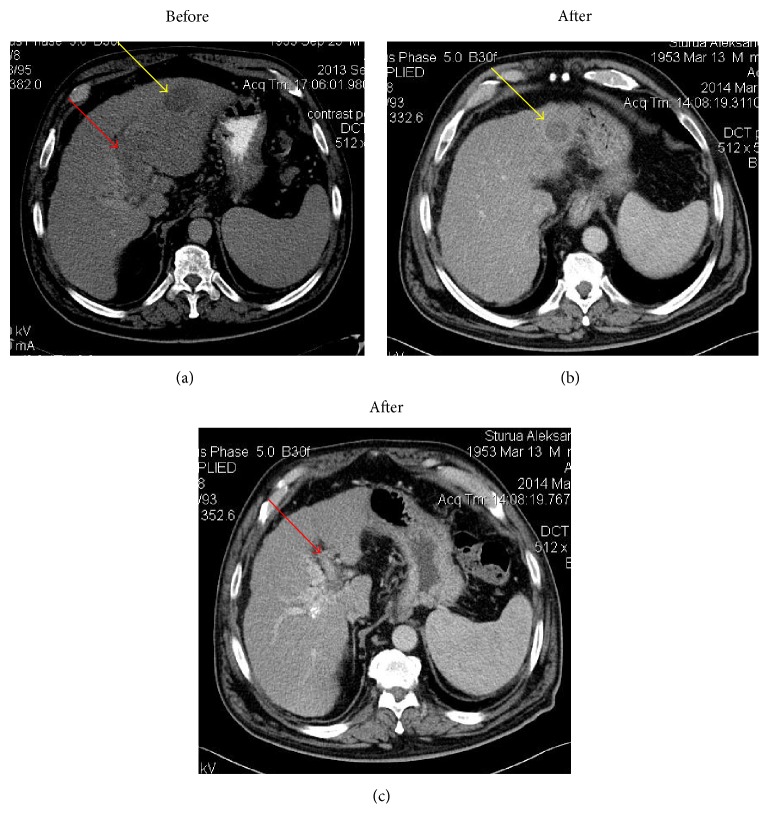
Comparison: CT before and in 5 months after VesOpen procedure. LPV (red arrow) is recanalized. The residual tumor is represented only by hypodense component, the size of which is decreased (yellow arrow).

CT revealed that the LPV, which had initially been absolutely closed with thrombus and was not processed on VesOpen procedure, was now recanalized (without any anticoagulation or thrombolytic therapy). The left lobe bulging, which has previously been evaluated as a big HCC, was reduced. Only sharply circumscribed, hypodense small mass was seen (Figures 3 and 4). The patient refused to undergo the scheduled TACE.

18 months after the VesOpen procedure, the patient was referred to the hospital for heart problems and has proceeded blood tests which showed the normalized blood coagulation values: PT-14.0; PT%-85.6; INR-1.12. Unfortunately, patient was lost for the subsequent follow-up.

## 3. Discussion

RFA is a safe and effective modality for the treatment of focal malignant diseases in solid organs and has been used to achieve localized tumor necrosis in solid neoplasms for many years [16, 17]. It delivers a high amount of thermal energy to target tissue with curative or palliative intent, which can be monitored by a real-time ultrasonography or a computed tomography.

During RF, the energy passes between the electrodes and biological tissues to cause coagulation of a selected area. The high-frequency alternating electric current applied through the electrodes results in rapid movement of intracellular ions in opposite directions. Ionic motion creates frictional forces that generate heat around the electrodes and eventually around the tissue surrounding the catheter.

Supporting the release of a wide spectrum of tumor antigens by in situ tumor destruction, RFA is considered to be a strong adjuvant for initiating antitumor immune responses by virtue overcoming immune tolerance and leading to the presentation of otherwise cryptic neoplastic antigen [18–20]. A tumor-specific T-cell activation following RFA has been documented in the nonreactive neoplasm-bearing host [18]. In humans, post-RFA HCC regression has been associated with the increased dendritic cell infiltration and consequent tumor-specific T-cell responses [21]. RFA of HCC was found to trigger a functional transient activation of myeloid dendritic cells associated with increased serum levels of TNF-alpha and IL-1 beta with a sustained antitumor immune response [20]. In addition, animals treated with subtotal RFA showed significant elevation in tumor-specific class I and II responses to a male minor histocompatibility (HY) antigens and tumor regression [22]. Thus, by providing several “danger” signals to the immune cells, RFA includes an active immunotherapeutic effect in cancer which demands further exploration; moreover that therapeutic vaccination of HCC is still an awaited approach [23].

Liver synthetic function is one of the important factors determining the treatment option in patients with primary liver cancer, thereby directly influencing the long term prognosis of these patients. Improving liver synthetic function in these patients makes them suitable for better treatment options. Partial or complete recanalization of the PV following RFA of tumor thrombus improves liver function and makes these patients suitable for better treatment options like transarterial chemoembolization (TACE), local ablative therapies, or systemic therapy with sorafenib. This potentially improves survival in this group of patients who were initially not suitable for any tumor-specific treatment due to poor liver function.

In the presented case, RFA and stenting were done on RPV, expecting to have the clinical effect by RPV recanalization. Interestingly, however, the follow-up examination showed that LPV was also recanalized and the left lobe tumor size decreased. As the LPV was not processed on VesOpen procedure, the only possible explanation of this effect is the antitumor immune response triggered by tumor thrombus RF processing.

## 4. Conclusion

In case of HCC with PVTT, RFA could be considered as an instrumental feasible procedure and the potential modulator of immune response against tumor.

## Figures and Tables

**Table 1:** Blood test results.

Test	Result	Normal range
Chemistry		
Albumin (g/L)	25	35–52
AST (u/L)	99.8	<40
ALT (u/L)	76.0	<41
GGT (u/L)	158	10–70
Hematology		
Platelets (nL)	70	150–400
Coagulation		
PT (sec)	16.8	11–15
PT (%)	61.9	70–105
INR	1.48	1–1.3
Immunology		
AFP (m/L)	31577	≤5.8

## Abbreviations

CT: Computed tomography
US: Ultrasound
PV: Portal vein
SMA: Superior mesenteric artery
RHA: Right hepatic artery
LHA: Left hepatic artery
RPV: Right portal vein
LPV: Left portal vein
PVT: Portal vein thrombosis
PVTT: Portal vein tumor thrombus
MDT: Multidisciplinary discussion
TACE: Transarterial chemoembolization
HCC: Hepatocellular carcinoma.
